# Brainstem tau pathology in Alzheimer’s disease is characterized by increase of three repeat tau and independent of amyloid β

**DOI:** 10.1186/s40478-017-0501-1

**Published:** 2018-01-03

**Authors:** Miho Uematsu, Ayako Nakamura, Momoko Ebashi, Katsuiku Hirokawa, Ryosuke Takahashi, Toshiki Uchihara

**Affiliations:** 1 Laboratory of Structural Neuropathology, Tokyo Metropolitan Institute of Medical Science, 2-1-6 Kamikitazawa, Setagaya-ku, Tokyo, 156-8506 Japan; 2 Department of Neurology, Kyoto University Graduate School of Medicine, Sakyo-ku, Kyoto, Japan; 3 The Japan Society for the Promotion of Science (JSPS), Chiyoda-ku, Tokyo, Japan; 4 Division of Neurology, Department of Medicine, Nihon University School of Medicine, Itabashi-ku, Tokyo, Japan; 5 Department of Pathology, Nitobe-Memorial Nakano General Hospital, Nakano-ku, Tokyo, Japan

**Keywords:** Alzheimer disease pathology, Tau isoform, Human brain, Virtual slide imaging, Brainstem

## Abstract

**Introduction:**

Alzheimer-type neuropil threads (NTs) and neurofibrillary tangles (NFTs) are comprised of either 4 repeat (4R)-tau, 3 repeat (3R)-tau, or a mixture of both. In the hippocampus, the number of NFTs, and the proportion of 3R tau progressively increases. If this preferential accumulation of 3R tau also occurs in the brainstem, it may be fundamentally related to progression of Alzheimer pathology.

**Methods:**

Midbrain and pontine sections of brainstems from 23 cases (Braak-NFT stages I/II: 8, III/IV: 8, and V/VI: 7) were double immunofluorolabeled for 4R and 3R tau. High-resolution (0.645 μm/pixel), in-focus snapshots were tiled to cover entire brain sections using a virtual slide system. Each lesion was classified by size (NT < 200 μm^2^ < NFT) and staining profile (3R/4R). In addition, the localization and quantity of amyloid β (Aβ) deposits were examined in adjacent sections for comparison with tau.

**Results:**

The data sets obtained from approximately 286 gigabytes of image files consisted of 847,763 NTs and 7859 NFTs. The proportion of 3R tau-positive NTs and NFTs in the midbrain, and 3R tau-positive NTs in the pons gradually increased with advancing NFT stages, while the proportion of 3R tau-positive NFTs in the pons was already elevated at early stages. Aβ deposits were absent at NFT stages I/II, and when present at later stages, their regional distribution was different from that of tau. These observations suggest that a progressive increase in the proportion of 3R tau occurs independently of Aβ deposits.

**Conclusions:**

This is the first quantitative analysis of NFTs and NTs in the human brainstem. We demonstrate that the proportion of 3R tau in the brainstem neurofibrillary changes increases with disease progression. Because this phenomenon is shared between the brainstem and the hippocampus, this increase may be fundamental to the pathogenesis of Alzheimer disease.

**Electronic supplementary material:**

The online version of this article (10.1186/s40478-017-0501-1) contains supplementary material, which is available to authorized users.

## Introduction

Neurofibrillary tangles (NFTs) and neuropil threads (NTs), collectively called the neurofibrillary changes, are pathological hallmarks of Alzheimer disease (AD), which exhibit a stereotypical pattern of hierarchical progression initiated around the hippocampus [5]. The extent of neurofibrillary changes correlates with the severity of dementia in AD [56]. Subcortical nuclei also exhibit Alzheimer-related neurofibrillary pathology [24, 29]. Probably prior to the limbic area, subcortical nuclei such as the dorsal raphe nucleus (DRN) and locus coeruleus (LC) develop neurofibrillary changes much earlier, because they are sometimes detectable under 30 years of age in subclinical phases [6, 19, 46]. However, it is not known how these brainstem lesions change along disease progression [2]. This prompted us to examine how neurofibrillary changes in the brainstem are similar to and different from those in the limbic areas with special attention to tau isoforms.

Neurofibrillary changes are immunoreactive mainly toward the hyperphosphorylated form of tau protein [20, 25]. Exon 9–12 of the tau gene, each containing imperfect repeat, encode a microtubule-binding domain, and inclusion or exclusion of exon 10 via alternative splicing generates isoforms with four repeats (4R tau) or three repeats (3R tau), respectively [17].

Paired helical filaments (PHFs) from the brains of AD consist of both 4R and 3R tau [18]. Neurofibrillary changes of neurofibrillary tangle-predominant dementia are also comprised of both 4R and 3R tau [31]. However, participation of each isoform is not random. At cellular level, each NFT is considered to undergo morphological changes from pretangles, NFTs, to the ghost tangles, and this morphological maturation has been shown to coincide with the transition from 4R tau predominant pretangles to 3R tau predominant ghost tangles. [21, 28, 34, 35, 54]. Interestingly, regional extent of each isoform is differently regulated; for example, 3R tau-positive lesions are abundant in the areas in which tau deposition begins early, such as parahippocampal area and subiculum, while 4R tau-positive lesions are dominant in the areas in which tau deposition begins later, such as CA4 [21, 28, 34, 35]. This regional gradient of isoform is maintained from the early to late stages of AD [21]. It is then hypothesized that chronological change of tau isoform, 4R for early pretangles vs. 3R for late ghost tangles, is orchestrated to generate regional gradient of tau isoforms throughout disease progression. If such orchestrated regulation of tau isoforms is fundamental to AD, it is plausible that similar phenomena are also at work in the brainstem.

Comprehensive quantitative analysis on the double immunofluorolabeled brainstem sections for 4R and 3R tau may clarify the tau isoform distribution in the brainstem lesions including the assessment of their colocalization. However, single-plane fluorescence image may suffer from unclear boundaries due to inaccurate focusing, which is highly problematic when quantifying the lesion sizes or morphologies. To overcome these problems, we changed conventional imaging strategy by introducing “extended focus imaging (EFI)”, which extracts pixels with maximal local contrast from multiple vertical planes and reconstructs a single in-focus image. With this strategy incorporated in the virtual slide system, it was possible to delineate even minute lesions such as NTs at whatever depth in the vertical planes of the section. While virtual slide imaging has been scarcely employed for quantitative analysis [47], it was only after these technical refinements that we were finally successful in extracting all the immunofluorolabeled lesions within the full range and depth of the section with clear contours, enrolling them all in the comprehensive quantitative analysis. Using this new method, we counted all the NTs and NFTs with different tau isoform profiles on postmortem brainstem sections from cases with different stages of neurofibrillary pathology in the present study.

With the advent of the same virtual slide system, we also attempted to map amyloid β (Aβ) deposits, another hallmark of Alzheimer-related pathologies, in the sections adjacent to those used for tau isoform quantification for direct comparison. Aβ deposition exhibits a hierarchical distribution from neocortex, gradually involving the brainstem after Thal amyloid phase 3 [51]. Aβ deposition plays a role in early-onset familial AD, caused by mutation of genes such as *APP* and *PSEN1* [16, 45]. Moreover, reports suggest that Aβ is essential for the initiation of synaptic and neuronal tau pathology [32, 60]. However, the correlation between the severity of dementia and degree of the Aβ accumulation is much weaker than that of neurofibrillary pathology [56]. Because few reports are available on the topographical distribution of Aβ in the brainstem [27] and no data are available on their possible relation to tau deposits in the brainstem, our novel approach may clarify possible relation between tau and Aβ deposition in the brainstem.

Here, our comprehensive quantitative analysis on double-immunofluorolabeled midbrain and pontine sections for 4R and 3R tau has demonstrated that the preferential increase of the proportion of 3R tau-positive neurofibrillary changes occurs with disease progression in the brainstem, but independently of Aβ deposition. Because this is a shared phenomenon between brainstem and hippocampus, it is fundamental to the progression of Alzheimer-related tau pathology.

## Materials and methods

### Subjects

Twenty three autopsied brains with different cortical NFT stages (Table 1, Braak and Braak’s NFT stages I/II: 8 cases, III/IV: 8 cases, V/VI: 7 cases) were enrolled in this study with informed consent and the approval of the ethics committee of Tokyo Metropolitan Institute of Medical Science and Nitobe-Memorial Nakano General Hospital. These cases have previously been anonymized and subjected to standardized neuropathological assessment at Tokyo Metropolitan Institute of Medical Science, including silver impregnations by Gallyas and Campbell-Switzer method, and DAB immunohistochemistry with anti-phospho-tau (Ser202, Thr205) antibody AT8 (Thermo Fisher Scientific K.K., Tokyo, Japan) of representative brain regions. The brainstem sections of all cases, and cortical sections of selected cases underwent DAB immunohistochemistry with anti-alpha-synuclein phospho (Ser129) monoclonal antibody (1:10,000, Clone pSyn#64, Wako, Japan). Diagnosis of NFT stage, Consortium to Establish a Registry for Alzheimer’s Disease (CERAD) neuritic plaque score, and the evaluation of Lewy body distribution and frequency were performed following the published criteria [5, 37, 38]. Cases with non-Alzheimer-type tau deposits (e.g. progressive supranuclear palsy, corticobasal degeneration, argyrophilic grain disease and Pick body disease) were excluded. Concomitant Lewy pathology was observed in case 1, 9, 13 and 23 (Table 1, Additional file 1).

**Table 1:** Characteristics of subjects

Case	Age at death	Sex	Braak’s cortical NFT stages	Lewy pathology	Brainstem NFT							Comparison of 3R tau % with 4R tau%				Formalin-fixed brain weight (g)	CERAD neuritic plaque score	Brainstem Aβ deposition										
					Midbrain				Pons									Midbrain					Pons					
					Superior colliculus	Periaqueductal gray	Linear raphe nucleus	Substantia nigra	Locus coeruleus	Dorsal raphe nucleus	Median raphe nucleus	Neuropil thread		NFT				Total	Superior colliculus	Periaqueductal gray	Linear raphe nucleus	Substantia nigra	Total	Locus coeruleus	Dorsal raphe nucleus	Median raphe nucleus	Reticular formation	Pontine nucleus
						Ventral/Dorsal		Medial/Lateral				Midbrain	Pons	Midbrain	Pons					Ventral / Dorsal		Medial/ Lateral						
1	74	M	I/II	+ (B)	+	+ / +	+	+/+	+	+	+	<	<	<	<	1310	0	–	–	–	–	–	–	–	–	–	–	–
2	81	M	I/II	–	+	+ / +	+	+/+	+	+	+	<	<	<	>	1455	A	–	–	–	–	–	–	–	–	–	–	–
3	71	F	I/II	–	+	++/++	+	+/++	+++	+	++	>	>	<	>	1240	0	–	–	–	–	–	–	–	–	–	–	–
4	77	M	I/II	–	+	+ / +	+	+/+	+	+	+	<	<	>	>	1350	0	–	–	–	–	–	–	–	–	–	–	–
5	78	M	I/II	–	+	+ / +	+	+/+	++	+	+	<	>	<	>	1410	A	–	–	–	–	–	–	–	–	–	–	–
6	87	M	I/II	–	+	+ / +	+	+/+	++	+	++	<	<	<	<	1375	B	–	–	–	–	–	–	–	–	–	–	–
7	68	M	I/II	–	+	+ / +	+	+/+	+	+	+	<	<	<	<	1390	0	–	–	–	–	–	–	–	–	–	–	–
8	63	M	I/II	–	+	+ / +	+	+/+	+	+	+	<	>	>	>	1515	0	–	–	–	–	–	–	–	–	–	–	–
9	86	F	III/IV	+ (B)	+	++ / +	+	+/++	+++	+	++	<	>	>	>	1190	B	–	–	–	–	–	–	–	–	–	–	–
10	93	M	III/IV	–	+	++ / +	+++	++/++	++	+	++	<	>	>	>	1430	B	++	++	- / +	–	–	++	++	–	–	–	–
11	100	F	III/IV	–	+	++ / +	++	+/++	+++	+	++	>	>	>	>	1150	A	–	–	–	–	–	+	+	–	–	–	–
12	83	F	III/IV	–	+++	++ / +	++	+/+	++	+	++	>	>	>	>	1100	B	++	++	+ / ++	–	+/−	+	+	–	–	–	–
13	86	M	III/IV	+ (T)	+	++ / +	+	+/+	+	++	+++	>	<	<	<	1270	B	+	+	–	–	–	+	+	–	–	–	–
14	92	F	III/IV	–	+	+ / +	+	+/+	+++	++	++	>	>	>	>	1130	B	+	+	–	–	–	–	–	–	–	–	–
15	95	F	III/IV	–	+	+ / +	+	+/++	+	+	+++	<	>	>	>	1260	C	++	++	- / +	–	+/−	–	–	–	–	–	–
16	80	M	III/IV	–	+	+ / +	+	+/+	+++	+	++	>	<	<	<	1400	B	+	+	–	–	+/−	–	–	–	–	–	–
17	82	M	V/VI	–	+++	+++ / +	++	++/++	++	++	+++	>	>	>	>	1185	C	+++	+++	+ / ++	+	+/−	+++	+++	+	++	+	+
18	93	M	V/VI	–	+	+++ / +	+++	++/+	+++	+++	+++	>	>	>	>	1080	C	+	+	- / +	–	+/+	+++	+++	+	++	+	–
19	94	F	V/VI	–	++	+++ / +	+++	++/+	+++	+	+++	>	>	>	>	1350	C	+++	+++	- / +	–	++/+	+	+	–	–	–	–
20	89	F	V/VI	–	+	+++ / +	+++	++/++	+++	++	+++	>	>	>	>	1300	B	++	++	+ / ++	+	–	+	+	–	–	–	–
21	102	F	V/VI	–	++	+++ / +	++	+/++	+++	+	++	<	<	>	>	1140	0	–	–	–	–	–	–	–	–	–	–	–
22	85	M	V/VI	–	++	+++ / +	+++	+++/++	+++	++	+++	<	<	<	<	1395	C	+++	+++	+ / ++	+	++/++	+++	+++	+	++	+	+
23	88	F	V/VI	+ (N)	+++	++ / +	+++	+++/++	+++	++	+++	>	>	>	>	1170	C	+++	+++	+ /+++	–	++/+	+++	+++	+	+	+	+

Concomitant Lewy pathology is described as brainstem-predominant (B), transitional (T) and neocortical (N), according to McKeith criteria [37]. The regional counts in the 1 mm^2^ microscopic field with maximal neurofibrillary tangle (NFT) or well-defined compact Aβ deposition density is graded as follows; − (absent): none in the field, + (sparse): 1–9/field, ++ (mild) 10–19/field, +++ (moderate): 20- /field. For the comparison of 3R tau (%) with 4R tau (%), **>** indicates that the proportion of 3R tau is greater than that of 4R tau, while **<** indicates that the proportion of 4R tau is greater than that of 3R tau, on each section

### Distinction of 4R and 3R tau by double immunofluorescence

**Fig. 1:**
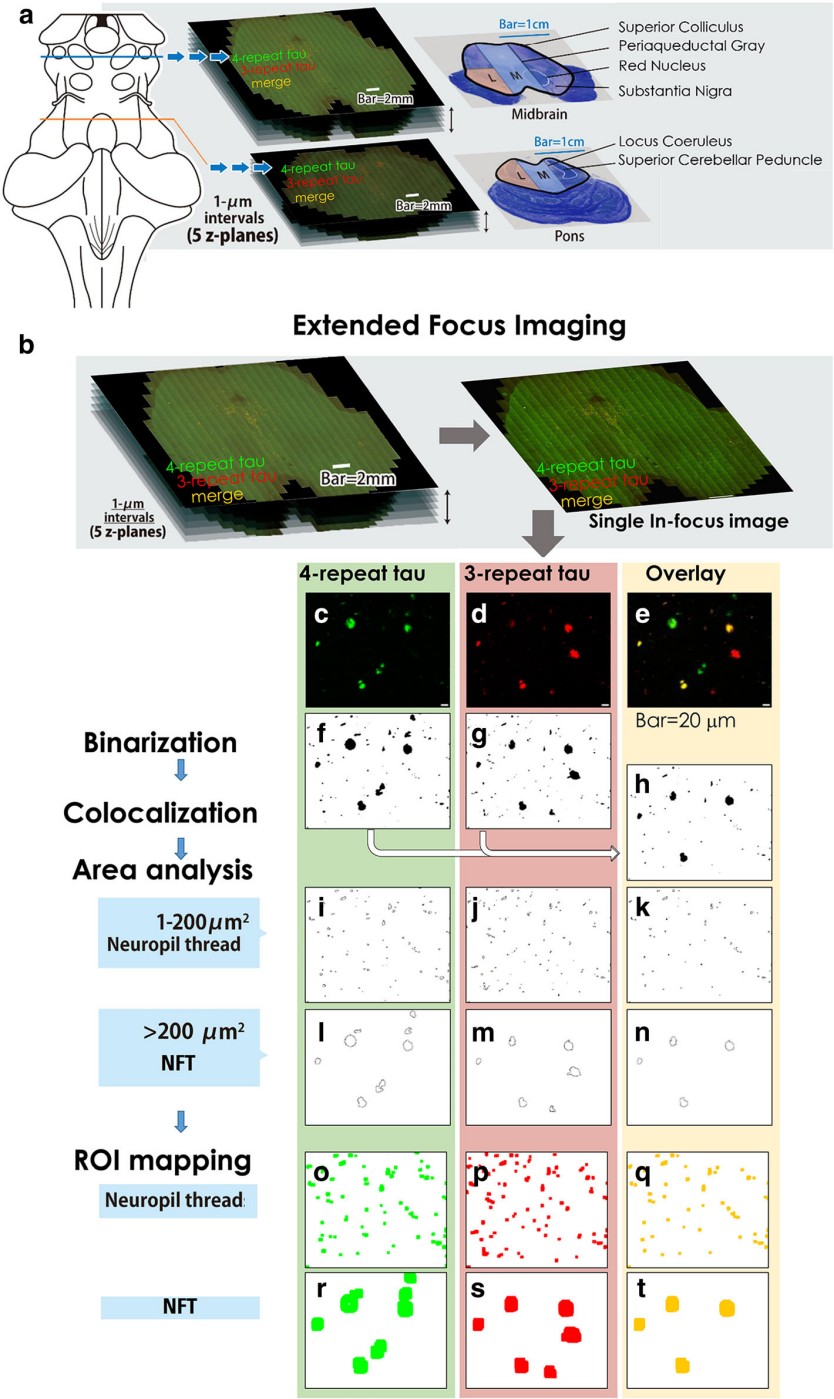
CENSUS (Complete ENumeration and Sorting for Unlimited Sectors) via immunofluorescence to count all neurofibrillary changes and sort everything by a virtual slide system. Image acquisition by virtual slide system and subsequent quantitative analysis and ROI mapping. (**a**) Image capturing at the coordinated anatomical levels. Midbrain sections included the superior colliculus (SC) and red nucleus (RN), and the pontine sections included the locus coeruleus (LC) and the superior cerebellar peduncles. The snapshots captured with 10 times objective lens in separate fluorescence channels were put together to cover the entire tectum and tegmentum. 5 vertical planes at 1 μm intervals were simultaneously captured. **(b**) The extended focus imaging (EFI) module picked up pixels with maximum local contrast to make a single in-focus image from 5 vertical planes. (**c**-**e**) Representative parts of the separate channels of the extended-focused images and their overlays are shown. Bar = 20 μm. (**f**-**h**) These images were binarized by Triangle algorithm (**f**, **g**), followed by binary colocalization analysis on ImageJ (h). (**i**-**n**) Particle analysis program sorted all immunolabels by size, which we defined as neuroil threads (NTs, area 1–200 μm^2^, **i**-**k**) and neurofibrillary tangles (NFTs, area > 200 μm^2^, **l**-**n**), with retrospective inspection into the original counterpart for confirmation. Outlines are shown. (**o**-**t**) ROI mapping of the immunolabels. The outlines of the ROIs selected in area analysis are uniformly exaggerated and colored (4R tau/*green*, 3R tau/*red*, colocalization/*yellow*) on ImageJ to abstract the immunolabels of NTs (**o**-**q**) and NFTs (**r**-**t**), as automatic immunolabel mappings

The autopsied brains were immersion-fixed in 4% formalin for 4 weeks. The brainstem was sliced perpendicular to the axis and embedded in paraffin. The levels of the sections were coordinated between cases by encompassing landmark anatomical structures (superior colliculus (SC) and red nucleus (RN) for the midbrain, and LC and superior cerebellar peduncles for the pons) (Fig. 1a). The 6-μm thick sections were deparaffinized for double immunofluorolabeling with antibodies against isoform-specific anti-4R tau antibody (rabbit polyclonal, Cosmo Bio Co, Tokyo, Japan), raised against amino acids 275–291 of human 4R tau, which is deamidated at N279 [10, 22], and anti-3R tau antibody (RD3, mouse monoclonal, Merck Millipore, Germany) [11]. To reduce diffuse cytoplasmic staining with the RD3, the sections were pretreated with 0.25% potassium permanganate for 15 min, 2% oxalic acid for a minute, and >99% formic acid for 30 min at room temperature, and autoclaved in 0.05 M citrate buffer for 20 min at 120 °C [53]. This pretreatment also improved immunolabeling with polyclonal anti-4R tau (data not shown). Sections were washed with phosphate-buffered saline (PBS) with 0.03% polyoxyethylene (10) octylphenyl ether (Triton X-100), blocked for 30 min in 5% normal goat serum/0.05% NaN_3_/PBS with 0.03% Triton X-100, and incubated with polyclonal anti-4R-tau antibody (1:3000) and RD3 (1:300), diluted in the blocking buffer at 4 °C for 4 days. To reduce autofluorescence of lipofuscin, sections were treated with Sudan Black B [44]. Quenching of the autofluorescence of lipofuscin was confirmed by spectral analysis (Additional file 2: Figure S1). Primary antibodies were labeled with Alexa 488 conjugated with anti-rabbit IgG (Molecular Probes, Oregon, USA, 1:200) and Alexa 568 conjugated with anti-mouse IgG (Molecular Probes, Oregon, USA, 1:200), respectively, diluted in PBS with 0.03% Triton X-100 overnight in the dark. Sections were mounted with buffered glycerol containing 0.1% *p*-phenylenediamine. For anatomical assessment, immediately adjacent sections were stained with Hematoxylin & Eosin and Klüver-Barrera (KB) methods.

### Diaminobenzidine (DAB) immunohistochemistry

For precise comparison of Aβ with tau deposits, neighboring sections from the same tissue blocks used for tau immunohistochemistry were subjected to Aβ/DAB immunohistochemistry. For the confirmation of the observation of 4R and 3R tau double-immunofluorolabeling, representative sections were also subjected to RD4 (mouse monoclonal anti-4R tau antibody, Merck Millipore, Germany) or RD3/DAB immunohistochemistry [11]. For Aβ, the sections were treated with > 99% formic acid for 5 min at room temperature. For RD4 or RD3, sections were pretreated as described in the section above [53]. After being treated with 1% H_2_O_2_ for 30 min, the sections were incubated in primary anti-Aβ mouse monoclonal antibody (Clone 6F/3D, M 0872; DAKO, Agilent Technologies, California, USA, 1:1000) at 4 °C for 2 days, RD4 (1:1000), or RD3 (1:3000) at 4 °C for 4 days, and biotinylated secondary anti-mouse antibody (BA-2000, Vector, California, USA, 1:1000) for 2 h. After subsequent incubation with streptavidin biotinylated horseradish peroxidase complex (ABC Elite, Vector, California, USA), color development was performed with DAB in the presence of imidazole and nickel ammonium chloride. They were examined by light microscopy, and the same virtual slide system used for tau deposits. For semi-quantitative grading of Aβ deposition, only the number of well-defined, compact Aβ deposition was counted, while ill-defined, fleecy deposition and dot-like deposition (smaller than approximately 1μm^2^) was not included in the count. The amount of well-defined, compact Aβ deposition in 1 mm^2^ microscopic field with maximum deposition density was graded as follows; − (None): no amyloid deposition in the field, + (Sparse): 1–9/field, ++ (Mild): 10–19/field, +++ (Moderate): 20- /field (Table 1).

### CENSUS (Complete ENumeration and Sorting for Unlimited Sectors) via immunofluorescence to count all neurofibrillary changes and sort everything by a virtual slide system (Fig. 1)

We obtained a virtual slide image covering full range and depth of the section at high resolution, and enrolled all the immunofluorolabels in the whole investigated area in comprehensive particle analyses. For this, we used a virtual slide system VS120 (Olympus, Tokyo, Japan) equipped with UPLSAPO 10 times objective lens (Olympus, Tokyo, Japan), a triple-band dichroic mirror unit (U-DM3-DA/FI/TX, Olympus), band path filter with peak emission wavelength at 518 nm (for 4R tau/Alexa 488) and 580 nm (for RD3/Alexa 568), a sensitive cooled charge-coupled device camera (ORCA-R^2^ C10600-10B, Hamamatsu Photonics, Shizuoka, Japan), and VS-ASW software (Olympus, Tokyo, Japan). Serial snapshots of a double-immunofluorolabeled section (4R tau/Alexa 488 and RD3/Alexa 568) were captured by VS120 on motorized stage with 10 times objective lens in separate fluorescence channels, and put together to make a seamless broad image, covering the whole tectum and tegmentum (Fig. 1a, Additional file 3: Figure S2). The resolution of each snapshot was 1376 pixels (horizontal) × 1038 pixels (vertical) at 0.645 μm/pixel with 10 times objective lens (original 8 bit). After subtracting the overlapping margins, the area per snapshot reduced to approximately 1138 pixels × 834 pixels (0.398 mm^2^/snapshot). 5 vertical planes at 1 μm intervals were simultaneously captured (Fig. 1a). To show all the immunolabels at full depth of the section with high accuracy, the EFI program on CellSens software (Olympus, Tokyo, Japan) extracted the pixels with maximum local contrast from the 5 vertical planes and made a single in-focus image (Fig. 1b, c-e), which minutely depicted even small threadlike lesions. These images were converted to big-tagged image file format, retaining the original resolution, to be further operated on ImageJ (NIH, Bethesda, Maryland, USA). The images were uniformly binarized in separate channels according to the threshold operationally defined by Triangle algorithm [59] on ImageJ (Fig. 1f, g). Colocalization analysis between binary images was subsequently performed with ImageJ plug-in (P. Bourdoncle, Institut Jacques Monod, Paris, France) (Fig. 1h). Particle analysis program of ImageJ showed particle size of each immunofluorolabel automatically, as well as X-Y coordinate, in separate fluorescence profiles. We defined particles with a 1–200 μm^2^ area as NTs (Fig. 1i-k), and with an area larger than 200 μm^2^ as NFTs (Fig. 1l-n). The results of the analyses were inspected against the original counterparts to ensure that each lesion was properly represented, and apparent contaminants (e.g. nonspecific staining of the vessel walls) were excluded from the count. For analysis of the regional differences, necessary parts were extracted from the virtual slide images. The regional counts in the 1 mm^2^ field with maximal NFT density was graded as follows; − (absent): no NFTs in the field, + (sparse): 1–9/field, ++ (mild) 10–19/field, +++ (moderate): 20- /field (Table 1). The results of the particle analyses were highlighted as regions of interest (ROIs) on ImageJ. The outline width of ROI depiction was uniformly widened and flattened onto a blank image file with same pixel size as the original. These abstracted outlines served as immunolabel mappings of NTs (Fig. 1o-q) and NFTs (Fig. 1r-t) in different tau-isoform profiles. Also, the virtual slide images of Aβ/DAB immunohistochemistry were captured with 10 times objective lens, and underwent thresholding by RGB values (R: 0–110, G: 60–150, B: 60–150) on CellSens software (Olympus, Tokyo, Japan). This procedure separated black-brown DAB labeling from reddish-brown neuromelanin and particle analysis highlighted the former as ROIs. The outline widths of these ROIs were uniformly widened and simplified on ImageJ to serve as the immunolabel mapping of Aβ deposition. We propose this virtual-slide based quantitative analysis of multifluorolabeled specimen as “Complete ENumeration and Sorting for Unlimited Sectors (CENSUS)” via immunofluorescence.

### Statistical analysis

All statistical analyses were performed using software R (version 3.2.3, R Foundation for Statistical Computing, Vienna, Austria.). *P* < 0.05 was considered significant. To find the differences in the means of data between cases, Fisher’s exact test or paired t-test with Bonferroni correction was performed. The counts of NTs and NFTs in each tau isoform, and their proportion to the total counts on the entire section were obtained. The proportional data underwent arcsine transformation prior to further analysis to make the data distribution closer to normal. Since arcsine of 1 equals 1.57, axis for the plot of arcsine-transformed proportion ranged from 0 to 1.57. The characteristics of individual cases were stratified into 3 subgroups as follows; age at death: young-old (ages 63–80 years, *n* = 7), middle-old (81–90 years, *n* = 9), and very-old (91–102 years, *n* = 7), NFT stages: I/II (*n* = 8), III/IV (*n* = 8) and V/VI (*n* = 7), CERAD plaque score: 0/A (*n* = 9), B (*n* = 8), C (*n* = 6), and the formalin-fixed brain weight (g): above 1350 (*n* = 8), 1200–1350 (*n* = 7), and below 1200 (*n* = 8). To assess whether there is a significant orderly increasing or decreasing trend along these stratifications, Jonckheere’s trend test was performed. To explore the best-fitting regression models of the plots, the model that yields the least Akaike’s Information Criterion was searched from quadratic (*y = ax*^*2*^ *+ bx + c*), linear (*y = bx + c* or *y = c*), exponential (*y = ab*^*x*^), and power (*y = ax*^*b*^) regression models (the formulas for all the regression models are available on request). The NFT stages (I/II, III/IV, V/VI), CERAD neuritic plaque score (0, A, B, C) and brainstem Aβ deposition (−, +, ++, +++) were converted to numeric variables from 0 to 3 [43]. Odds ratio and 95% confidence intervals for the percentages of sections with greater proportion of 3R tau than 4R tau during the advancement of cortical NFT stages (from I/II, III/IV to V/VI) were calculated by a univariate binomial logistic regression model (Additional file 1). In the box plots, the box represents the 25th and 75th percentiles, the horizontal line in the box represents the median, whisker show the 10th and 90th percentiles, and the white circles represent the outliers. Lines with asterisk indicate statistically significant differences.

## Results

### Topographical distribution is similar between NTs and NFTs, and between 4R and 3R tau (Fig. 2)

**Fig. 2:**
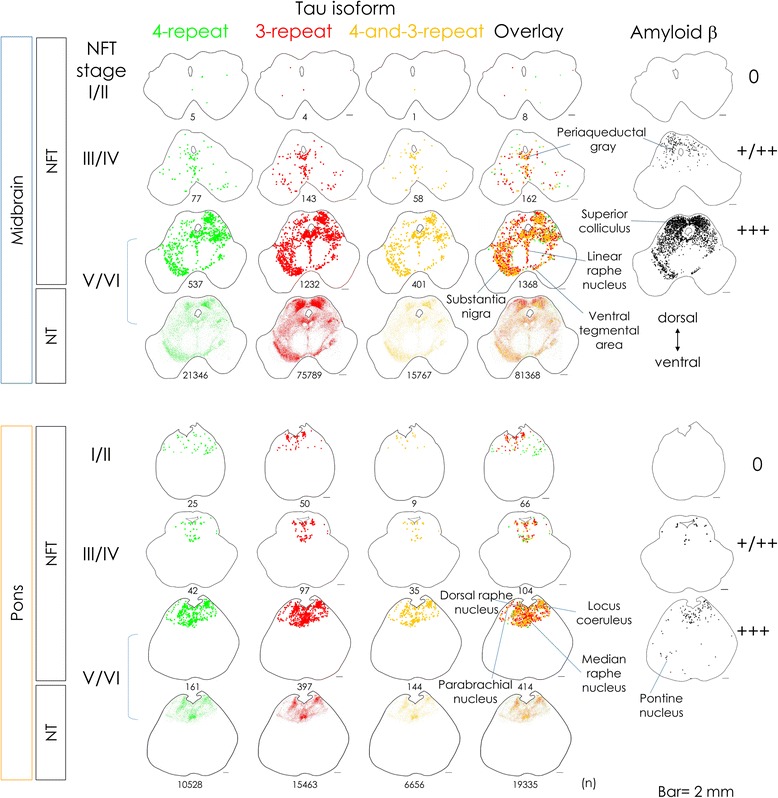
Topographical distribution is similar between NTs and NFTs, and between 4R and 3R tau. Representative immunolabel mapping of the tau deposition in separate isoform profiles (4R tau-positive/*green*, 3R tau-positive/*red*, 4R/3R tau both-positive/*yellow*), overlays, and amyloid β (Aβ, *black*). The number indicates the sum of immunolabels on each section. From top to bottom: the midbrain of case 5, 10, and 23, and the pons of case 3, 10, 23, with cortical NFT stages I/II, III/IV, V/VI, and the amount of local Aβ deposition of 0, +/++, +++, respectively. Bar = 2 mm. The dot sizes are irrelevant to the scale. Topographical distribution did not differ considerably between NFTs and NTs, and between tau isoform profiles. However, tau and Aβ deposits were different in their distribution, i.e.; at the periaqueductal gray (PAG, ventral-half vs. dorsal-half dominance in case 10), linear raphe nucleus (LRN), and dorsal raphe nucleus (DRN) (abundant tau deposition vs. few Aβ in cases 10 and 23)

Midbrain and pontine sections from 23 cases with different NFT stages were double-immunofluorolabeled for 4R and 3R tau (Table 1). The neurofibrillary changes were present in midbrain and pons of all the investigated cases (Table 1). We captured 300,860 snapshots (approximately 0.398 mm^2^ per snapshot after subtracting the overlapped area) of these sections in total, which were 30,086 snapshots on XY planes with 5 vertical planes in 2 fluorescence channels, by the virtual slide system (detailed in Additional file 1). The investigated area was approximately 120 cm^2^ in total. We performed comprehensive quantitative analyses on these images as described above (Fig. 1). The data sets obtained from approximately 286 gigabytes of image files consisted of 847,763 NTs (602,839 in the midbrain, 244,924 in the pons) and 7859 NFTs (4948 in the midbrain, 2911 in the pons). Each data retained the information of the location and tau isoform profile of the particle. Based on the results of quantitative analyses, immunolabel maps of the NTs and NFTs in different tau isoform profiles were operationally drawn (representative maps in Fig. 2).

The topographical distribution was similar between NFTs and NTs (Fig. 2, bottom two rows of midbrain and pons), and between 4R and 3R tau (Fig. 2, left four columns). The neurofibrillary changes were accentuated at the periaqueductal gray (PAG, ventral half-dominant), linear raphe nucleus (LRN), ventral tegmental area, and substantia nigra (SN) of the midbrain, and the LC, DRN, median raphe nucleus (MRN), and parabrachial nucleus of the pons (Fig. 2). Immunolabel maps of Aβ were also operationally drawn (Fig. 2, right column). Aβ deposits were accumulated at the SC, PAG (dorsal half-dominant), SN (medial half-dominant), LC, reticular formation (RF), and MRN. Topographical differences between tau and Aβ deposits will be described later in detail.

### Total counts of neurofibrillary changes increase with disease progression (Fig. 3)

**Fig. 3:**
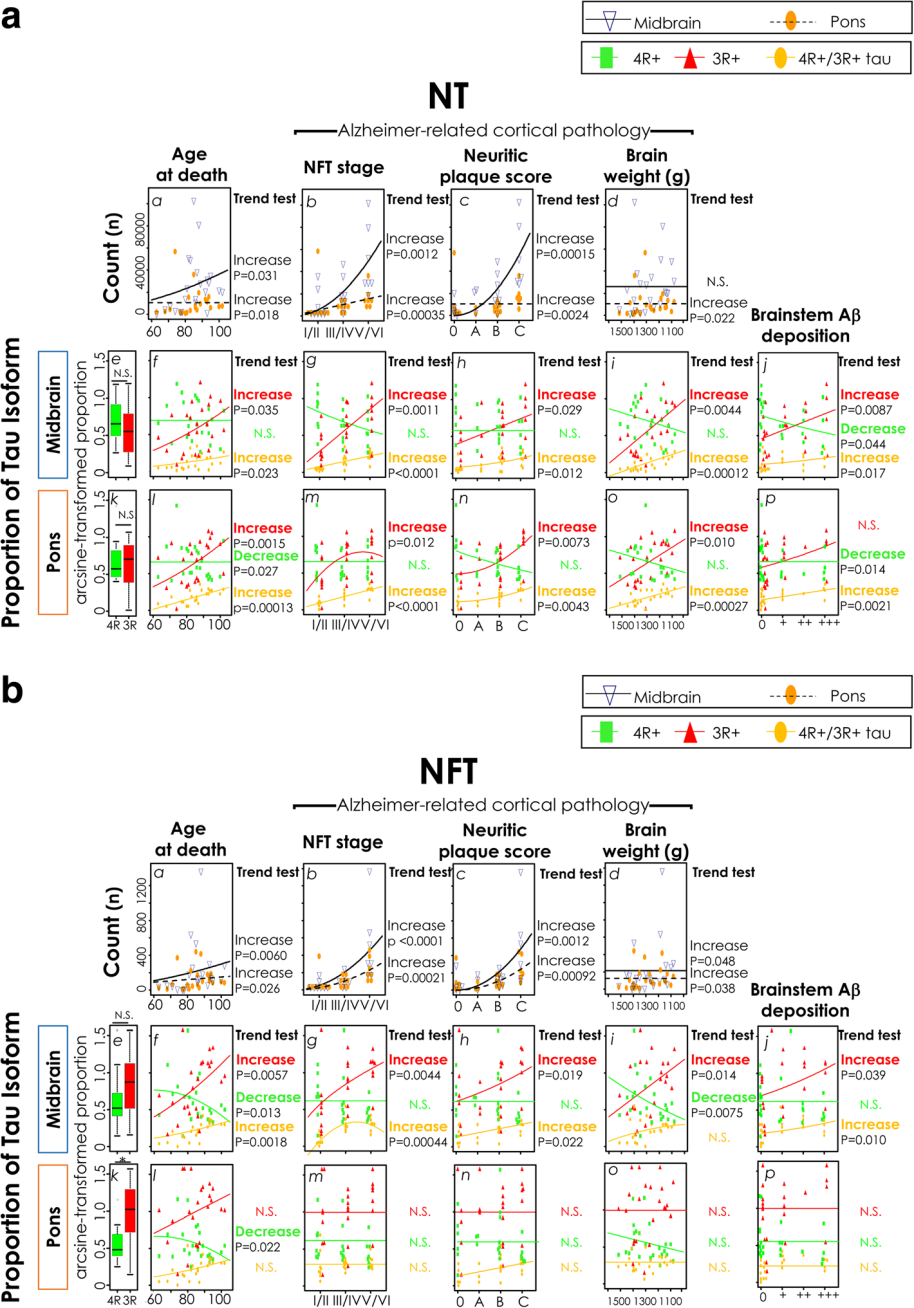
The proportion of 3R tau-positive midbrain NFTs progressively increases with disease progression, while that of pontine NFT is persistently dominant over 4R tau. (**a** *a*-*d*, **b** *a*-*d*) The plots and best-fitted regression curves/lines of total counts of the NTs (**a** *a*-*d*) and NFTs (**b** *a*-*d*) against age at death, and Alzheimer-related cortical pathologies of the individual cases: i.e., cortical NFT stage, CERAD neuritic plaque score, and brain weight (descending order). Midbrain (*navy triangle*) and pons (*orange oval*). P-values of the Jonckheere’s one-sided test for the orderly increasing or decreasing trend are indicated at the *right* side of the regression models when *p* < 0.05. N.S.: not significant. The total counts significantly increased with advancing age and Alzheimer-related cortical pathologies, with minor exception (**a** *d*, the count of the midbrain NTs against brain weight reduction, Jonckheere’s trend test, *p* = 0.054). (**a** *e, k*, **b** *e, k*) *Box plots* of the arcsine-transformed proportion of 4R (*green*) and 3R (*red*) tau-positive lesions. Only pontine NFTs showed significantly higher overall mean of the proportion of 3R tau-positive lesions than that of the 4R tau (*p* = 0.0093 (*), paired t-test with Bonferroni correction). (**a** *f*-*j*, *l*-*p*, **b** *f*-*j*, *l*-*p*) Plots of arcsine-transformed proportion (4R tau/*green rectangle*, 3R tau/*red triangle*, 4R/3R tau both-positive/*yellow oval*) against age at death, cortical NFT stage, CERAD neuritic plaque score, brain weight (in inverse order), and the degree of local Aβ deposition at the midbrain and pons, with best-fitted regression models. *P*-values of Jonckheere’s trend test are noted when *p* < 0.05 (from *top* to *bottom* on the *right side* of each panel; 3R tau-positive/*red*, 4R tau-positive/*green*, 4R/3R tau both-positive/*yellow*). The proportion of 3R tau-positive and/or 4R/3R tau both-positive lesions showed significant increasing trends with advancing age and Alzheimer-related cortical pathologies, as well as local Aβ deposition, except for those of pontine NFTs, which remained stably elevated (**b** *l*-*p*)

We assessed whether the total counts of neurofibrillary changes in the brainstem increase with disease progression (Fig. 3a *a-d*, b *a-d*). Jonckheere’s one-sided test for increasing trend generally revealed significant increasing trends of the total counts of NTs and NFTs, with advancing age and Alzheimer-related cortical pathologies; i.e., cortical NFT stage, CERAD neuritic plaque score, and brain weight reduction (Fig. 3a *a-d*, b *a-d*, *p* < 0.05), apart from a minor exception (Fig. 3a *d*, the count of the midbrain NTs against brain weight reduction, Jonckheere’s trend test, *p* = 0.054).

### The proportion of 3R tau-positive midbrain NFTs gradually increases to be dominant along disease progression, while that of pontine NFTs is persistently elevated (Fig. 3)

To see which of the tau isoforms contributes to the increase of total counts of neurofibrillary changes with disease progression, we calculated the proportion of 4R and 3R tau-positive neurofibrillary changes to the total, and compared their overall means by paired t-test (Fig. 3a *e, k*, b *e, k*). The overall mean of the proportion of 3R tau-positive pontine NFTs (Fig. 3b *k*, red box plot) was significantly higher than that of 4R tau (Fig. 3b *k*, green box plot, paired t-test with Bonferroni correction, *p* = 0.0093), while the difference was not significant for the NTs of the midbrain and pons (Fig. 3a *e, k*) and the NFTs of the midbrain (Fig. 3b *e*) (paired t-test with Bonferroni correction, *p* = 1.0, 1.0 and 0.68, respectively).

We next assessed whether the proportion of 4R and 3R tau-positive NTs and NFTs alter with disease progression at the midbrain and pons. Jonckheere’s one-sided test for increasing or decreasing trend (detailed in method) revealed that the NTs of the midbrain and pons and the NFTs of the midbrain showed significant increasing trends in the proportion of 3R tau-positive lesion with advancing age (Fig. 3a *f, l*, b *f*), NFT stage (Fig. 3a *g, m*, b *g*), CERAD neuritic plaque score (Fig. 3a *h, n*, b *h*), and reducing brain weight (Fig. 3a *i, o*, b *i*). Furthermore, in the subgroups of very-old age (age 91-), advanced NFT stages (V/VI) and low brain weight (below 1200 g), paired t-test revealed that the mean of the proportion of 3R tau-positive midbrain NFT was significantly higher than that of 4R tau (paired t-test with Bonferroni correction, *p* = 0.0017, 0.033, 0.0017, respectively, detailed in Additional file 1), indicating that the proportion of 3R tau-positive midbrain NFTs progressively increases and dominates over that of 4R tau in the advanced phase. The proportion of 4R/3R tau both-positive lesion also showed significant increasing trend for NTs of the midbrain and pons (Fig. 3a *f-j*, *l-p*) and the NFTs of the midbrain (Fig. 3b *f-j*), with advancing age and Alzheimer-related pathologies, with a minor exception (midbrain NFT against brain weight reduction, Fig. 3b *i*, Jonckheere’s trend test, *p* = 0.061).

On the other hand, the pontine NFTs did not show any increasing or decreasing trend for the proportion of 3R tau or 4R/3R tau both positive lesions (Fig. 3b *l-p*) on Jonckheere’s trend test. Together with the result of paired t-test (Fig. 3b *k*), this result indicated that the proportion of 3R tau-positive pontine NFTs were persistently elevated from younger age and early phase of the disease.

Taken together, these findings indicated that, along the increase in the total count of neurofibrillary changes, the proportion of 3R tau-positive midbrain NFTs increased with disease progression and became significantly higher than that of 4R tau in advanced disease subgroups, while the proportion of 3R tau-positive pontine NFTs remained stably elevated from the early phase.

### RD4 and RD3 DAB immunohistochemistry (Fig. 4)

The adjacent sections taken from the same tissue blocks as the representative sections depicted in Fig. 2 were subjected to single DAB immunohistochemistry with RD4 or RD3 antibodies to assess the reproducibility of the observation by immunofluorescence. DAB immunohistochemistry of these representative sections was in line with the finding based on immunofluorescence that the proportion of 3R tau positive NFTs in the midbrain increased with advancing NFT stages, while the proportion of 3R tau positive NFTs in the pons was high already in early stages.

**Fig. 4:**
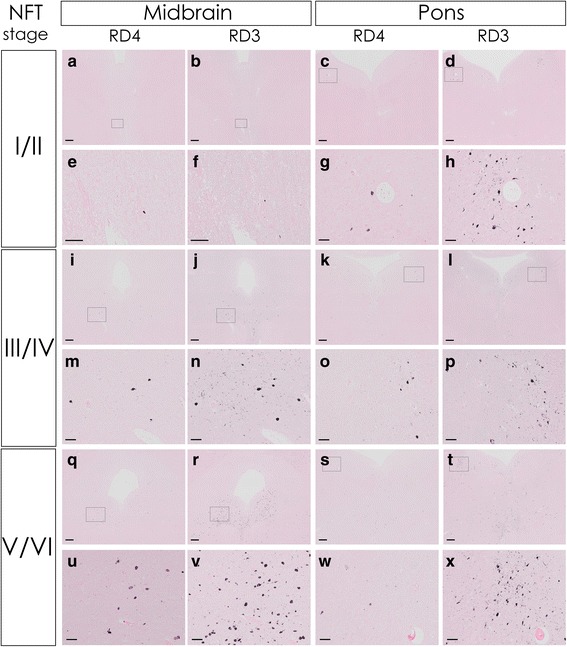
RD4 and RD3 DAB immunohistochemistry. Representative adjacent sections of the same cases as those shown in Fig. 2 underwent DAB immunohistochemistry with RD4 or RD3. From top to bottom: the midbrain of case 5, 10, and 23, and the pons of case 3, 10, 23, with cortical NFT stages I/II, III/IV, V/VI, respectively. Insets in the images above (**a**-**d**, **i**-**l**, **q**-**t**) correspond to the area shown in the images below (**e**-**h**, **m**-**p**, **u**-**x**) for each section. The results of DAB immunohistochemistry were in line with the findings based on immunofluorescence that the proportion of 3R tau positive NFTs in the midbrain increased with advancing NFT stages while the proportion of 3R tau positive NFTs in the pons was high already in early stages. Scale bar = 500 μm (**a**-**d**, **i**-**l**, **q**-**t**), 100 μm (**e**-**h**, **m**-**p**, **u**-**x**)

### Topographical distribution of neurofibrillary changes on the midbrain and pontine sections (Fig. 5)

**Fig. 5:**
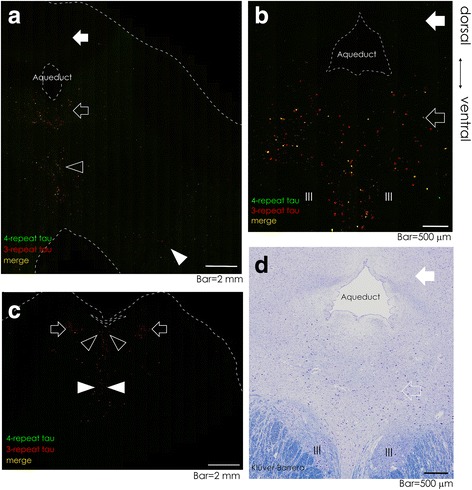
Topographical distribution of neurofibrillary changes at the midbrain and pons. Representative virtual slide images double-immunofluorolabeled with anti-4R (*green*) and 3R (*red*) tau, with colocalization (*yellow*) on overlays, of the midbrain (**a**, **b**, case 18 and 10, respectively) and pons (**c**, case 18). At the midbrain, the neurofibrillary changes were accentuated at the ventral PAG (**a**, **b**, *open arrow*), LRN (**a**, *open arrowhead*), and substantia nigra (SN, **a**, *solid arrowhead*). The neurofibrillary changes often affected the ventral half of the PAG (**a**, **b**, *open arrows*) more severely than the dorsal half (**a**, **b**, *solid arrows*). By comparison to KB-stained section (**d**, case 10, nearby section to **b**), the lesions in the ventral PAG coincided with the area where the large neurons of DRN were present (**d**, *open arrow*). The oculomotor nuclei (**b**, **d**, III) were not involved with neurofibrillary changes. At the pons, the neurofibrillary changes were accentuated at the LC (**c**, *open arrows*), DRN (**c**, *open arrowheads*), and median raphe nucleus (MRN, **c**, *solid arrowheads*). Scale bar = 2 mm (**a**, **c**), and 500 μm (**b**, **d**)

We next aimed to clarify the topographical distribution of neurofibrillary changes on the midbrain and pontine sections from 23 cases (Table 1, Fig. 5). In 80% of the cases with NFT stages III-VI (Table 1, 12 out of 15 cases), neurofibrillary changes affected the ventral half of PAG more severely (Fig. 5a, b, open arrows) than the dorsal counterpart (Fig. 5a, b, solid arrows, *p* = 0.000011, Fisher’s exact test). By comparison to KB-stained section (Fig. 5d, nearby section of Fig. 5b), the ventral predilection sites of neurofibrillary changes at the PAG were rich in large neurons of DRN (Fig. 5d, open arrow), while the dorsal counterparts were rich in smaller neurons (Fig. 5d, solid arrow). At the midbrain (Fig. 5a, Table 1), the neurofibrillary changes were also accentuated at the LRN (Fig. 5a, open arrowhead), ventral tegemental area, and SN (Fig. 5a, solid arrowhead). At the pons, the neurofibrillary changes were accentuated at the LC (Fig. 5c, open arrows), DRN (Fig. 5c, open arrowheads), MRN (Fig. 5c, solid arrowheads), and parabrachial nucleus (also Table 1).

### Progressive accumulation of Aβ deposits is not topographically parallel to tau deposits (Fig. 6)

**Fig. 6:**
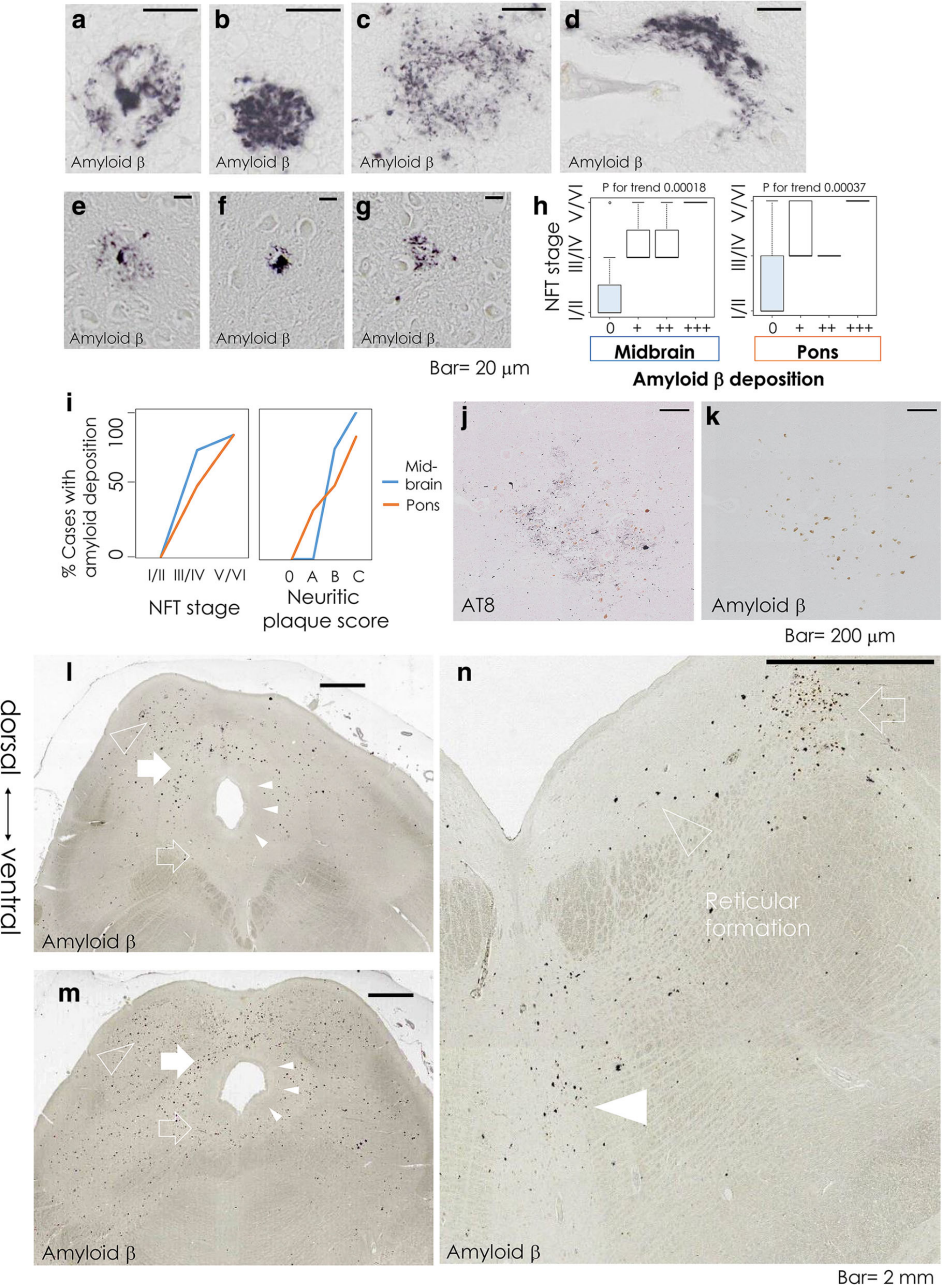
Progressive accumulation of Aβ deposits is not topographically parallel to tau deposits. **a**-**g** Representative midbrain (**a**-**d**, case 23, SC) and pontine section (**e**-**g**, case 23, pontine nucleus), stained with Aβ/DAB. As Iseki et al. have described [27], Aβ deposits showed various morphologies; e.g., amyloid plaques type 1 (**a**, **e**, *‘amyloid core with surrounding processes’*), type 2 (**b**, **f**, *‘amorphous amyloid with surrounding processes’*), and type 3 (**c**, **g**, *‘ill-defined aggregation of the fine processes’*). Fleecy amyloid deposits [50] surrounded a capillary wall (d). Scale bar = 20 μm. (**h**) Box plots of the cortical NFT stages in different degrees of local Aβ deposition at the midbrain (*left* panel) and pons (*right* panel). **i** Percentages of the cases with Aβ deposition at the midbrain (*blue*) and pons (*orange*) with advancing NFT stages (*left* panel) and CERAD neuritic plaque score (*right* panel). **j**, **k** DAB staining of AT8 (**j**) and Aβ (**k**) immunohistochemistry at the LC (case 21) showed absence of Aβ in the presence of tau deposition in this case. Bar = 200 μm. **l**-**n** Aβ/DAB-stained sections of the midbrain (**l**, **m**, cases 10 and 23, respectively) and pons (**n**, case 22) showed dorsal predilection of Aβ deposition at the PAG (l, *solid arrow*), which was dissociated from the ventral predilection of tau deposition (Fig. 5a, b, *open arrows*). The SC (**l**, **m**, *open arrowheads*) showed abundant Aβ deposition. The subnucleus medialis (**l**, **m**, *solid arrowheads*) was free of Aβ depositions. Bar = 2 mm. LC (**n**, *open arrow*), MRN (**n**, *solid arrowhead*) and reticular formation (**n**) showed involvement with Aβ deposition. Lesions in the DRN (**n**, *open arrowhead*) were only sparsely detected. Bar = 2 mm

Next, we compared the topographical distribution of Aβ deposits to that of tau on neighboring sections at the midbrain and pons. Aβ deposits at the midbrain (Fig. 6a-d) and pons (Fig. 6e-g) showed various morphologies. Fleecy amyloid deposits [50] surrounded some of the capillary walls (Fig. 6d). The amount of Aβ deposits was associated with increasing trends of NFT stages (Jonckheere’s trend test, *p* = 0.00018 and 0.00037, for midbrain and pons, respectively, Fig. 6h). However, only 57% of investigated cases showed Aβ deposits at either or both the midbrain and pons (Table 1, Fig. 6i, 0% of the cases with NFT stages I/II, 87.5% of stages III/IV and 86% of stages V/VI). For example, case 21, with cortical NFT stages V/VI and CERAD neuritic plaque score 0 (Table 1), exhibited AT8-positive neurofibrillary changes at the midbrain and pons (Fig. 6j, LC), but no Aβ deposits on the neighboring sections (Fig. 6k).

When present, the SC (Fig. 6l, m, open arrowheads) was the most severely affected region of all investigated areas by Aβ deposition (Table 1), and the predilection sites included the LC (Fig. 6n, open arrow), MRN (Fig. 6n, solid arrowhead), and RF (Fig. 6n). Lesions in RN and pontine base were only sparsely detected even in advanced cases. The subnucleus medialis of the PAG (Fig. 6l, m, solid arrowheads), which surrounds the aqueduct, did not show Aβ deposition in the investigated cases.

The predilection sites of Aβ deposition differed considerably from those of tau (Table 1, Fig. 2) at the PAG, DRN, LRN, and SN, as described below. The Aβ deposition affected the dorsal PAG more severely (Fig. 6l, solid arrow) than the ventral counterpart (Fig. 6l, open arrow) in 9 out of 9 cases with any deposition therein (*p* = 0.000041, Fisher’s exact test). This dorsal predilection of Aβ at the PAG dissociated from the ventral predilection of tau deposits (Fig. 5a, b, open arrows). Also, at the DRN and LRN, where tau deposits were abundant, the Aβ deposits were rarely detected (Table 1). Finally, medial half of the SN was affected by the Aβ deposition more severely than the lateral half in 6 out of 8 cases with any deposits therein (75%, *p* = 0.0070, Fisher’s exact test), while the amount of tau deposits was not significantly different between medial and lateral SN (*p* = 1.0, Fisher’s exact test).

Taken together, the Aβ deposition at the midbrain or pons was absent in all the cases with NFT stages I/II, although gradually increased to be present in 86% of stages V/VI. When present, the predilection sites of Aβ deposition included the SC, PAG (dorsal half-dominant), SN (medial half-dominant), LC, RF, and MRN. On the other hand, the ventral half of the PAG, DRN and LRN, which were the areas in which neurofibrillary changes were abundant, were much less intensely affected by Aβ deposition. These differences in distribution suggested that the tau and Aβ deposition may occur independently of one another at least to some extent.

## Discussions

In this study, we performed virtual-slide based comprehensive quantitative analyses on double-immunofluorolabeled sections of midbrain and pons for 4R and 3R tau, and clarified that the proportion of 3R tau-positive midbrain NFTs progressively increased (Fig. 3b *e-i*) and dominated in the advanced disease phase, while that of pontine NFT persistently dominated over 4R tau (Fig. 3b *k-o*). The obtained results suggested a possibility that the dominant immunoreactive epitopes of tau isoform is changing along disease progression in neurofibrillary changes, even after their formation.

For tau-isoform specific immunolabeling, two monoclonal antibodies, RD3 and RD4, have been used as the standard. However, RD3 and RD4 are both monoclonal antibodies raised in mouse, which hampers their distinction if anti-mouse secondary antibodies are used for their fluorodetection. Double labeling with two unconjugated primary antibodies raised in the same host species is difficult to achieve, and requires unconventional procedures. To circumvent this cross talk, our group performed double immunofluorolabeling with RD3 and RD4 in the previous studies by a combination of tyramide signal amplification (TSA) of the hyper-diluted RD3, followed by conventional immunofluorolabeling of the RD4 [21, 54]. Although this double labeling by the TSA method enabled precise quantification of RD3/RD4 labeling on NFTs on hippocampus [21], the staining procedure is rather complex. Recently, a rabbit polyclonal antibody raised against 4-repeat tau, which is deamidated at asparagine residue 279, has been reported to strongly stain tau lesions in the Alzheimer’s disease brain without cross reaction with 3R tau [10]. Introduction of this rabbit polyclonal antibody against 4R tau for the double immunofluorolabeling along with the mouse monoclonal antibody RD3 is much more simple and straightforward, because these antibodies are raised in different host species. The amount of DAB immunolabeling with polyclonal anti-4R tau (1:30,000) was equivalent to or slightly greater than the amount of immunolabeling with monoclonal RD4 (1:1000, Additional file 4: Figure S3).

The prevalence of each isoform can be precisely demonstrated by immunohistochemistry via brightfield chromogenic detection such as DAB [28, 34, 35]. However, DAB labeling is convincing if only single epitope (3R or 4R tau) is evaluated. Comparison of adjacent sections may identify double labeled lesion (3R and 4R tau) only if the targets are large enough as NFTs to be included in both sections. In this study, however, we also aimed to examine NTs for their sizes and isoform profiles (3R and 4R tau). Because the threads are much smaller than NFTs, exact identification of isoform profile (3R only, 3/4 R both positive, 4R only) on such small target is not feasible through comparison of adjacent sections, or bright-field dual-color chromogenic labeling. Thus, colocalization of 4R and 3R tau isoforms on the thread is accurately distinguishable only with multi-immunofluorolabeling [21, 23, 54].

### Complete ENumeration and Sorting for Unlimited Sectors (CENSUS) via immunofluorescence

Because our CENSUS approach standardizes the acquisition condition of fluorescence microscopic images and operationally picks up all the immunopositive lesions contained in the histological sections (Fig. 1), the data sets, once obtained, can be arbitrarily processed for size quantification (Fig. 1), mapping based on XY coordinates (Figs. 1, 2), calculations of the proportion of isoform (Fig. 3), and their possible relation to disease progression can be demonstrated (Fig. 3). This CENSUS fundamentally changed conventional morphometric approaches, usually calculating local density of predefined ROIs, to test whether the results corroborate the predefined working hypothesis. Moreover, local density of a lesion may be influenced by the atrophy of the target structure and overall neuronal loss [52]. For example, an increase in the lesion density may be accentuated if the entire structure is atrophied, even when the count number remained relatively unchanged. Because CENSUS approach picks up all the lesions in the section, interpretation is much more straightforward than conventional ROI-based approach.

### Progressive dominance of 3R tau lesions in the postmortem brainstem

Previous reports showed that the NFTs matures morphologically from 4R tau dominant pretangles to 3R tau dominant ghost tangles in the hippocampus, and that the proportion of 3R tau-positive neurofibrillary changes was higher in the hippocampal subregions with advanced neurofibrillary pathology than those involved in later stage [21, 28, 34, 35, 54]. Thus, it is hypothesized that this tau-isoform transition during the morphological maturation of the NFT is orchestrated to form the regional progression of 3R tau dominance in the hippocampus along the perforant path containing unidirectional hippocampal circuitry, beginning in the entorhinal and transentorhinal cortices, subsequently progressing to the subiculum and CA1, and further to CA 3–4 [21]. In the previous studies, however, the effect of disease progression on tau isoform prevalence was not fully evaluated [21, 28, 34, 35].

In this study, we enrolled sufficient number of samples to evaluate the effect of disease progression, and by employing CENSUS approach, we clarified that the proportion of 3R tau-positive NFTs in the midbrain and the NTs in the midbrain and pons gradually increased with advancing Alzheimer-related cortical pathology (Fig. 3a *f-i*, *l-o*, b *f-i*). This gradual increase in the proportion of 3R tau probably explain why there was not a significant difference between the overall means of the proportion of 4R and 3R tau in these neurofibrillary changes (Fig. 3a *e*, *k*, b *e*, paired t-test). On the other hand, the overall mean of the proportion of 3R tau-positive pontine NFT was significantly higher than that of 4R tau (Fig. 3b *k*) and it was stably elevated along disease progression (Fig. 3b *l-o*, Jonckheere’s trend test), indicating that the proportion of 3R tau-positive NFTs was persistently dominant in the pons but not in the midbrain. This difference does not necessarily indicate that the mechanism of tau deposition is different between pons and midbrain. Rather, this difference is explained if pontine neurons are liable to develop neurofibrillary changes from earlier stage than midbrain neurons. It is then expected that similar progressive dominance in 3R tau may be detectable if pontine samples from younger individuals are included, which is a subject for future studies. If regional gradient of isoform around hippocampus is oriented along a defined major circuity such as performant path, what kind of circuitries in the brainstem are responsible for the gradient? Because neuroanatomical connections in the brainstem are much more complex [4, 39, 40, 42] than that of the hippocampus, it is practically impossible to identify possible candidate circuitries in the brainstem, if any, that may account for such gradient. It is also possible that isoform regulation could be independent of the circuit and each neuron may regulate tau isoforms independently of each other, which needs fundamental reconsiderations in the future studies. Although preceding studies have suggested that the earliest neurofibrillary lesions are detected in the brainstem [6, 19, 46], it is difficult to directly compare the extent of 3R tau dominance between different anatomical structures, because the densities and characteristics of the underlying neuronal population are quite different. Therefore, we focused on the trend of increase for the proportion of RD3 at the coordinated horizontal anatomical levels between cases, rather than direct comparison between the data of the midbrain and pons or between the data of the brainstem and the hippocampus (Additional file 5: Figure S4), and did not attempt to show that brainstem tau lesions develop prior to hippocampus in this study. Rather, we demonstrated that the progressive accumulation of 3R tau over 4R tau is shared between brainstem and the hippocampus.

Biochemical evaluation for 3R and 4R tau by immunoblotting may be potentially interesting and helpful in the future studies. However, considering the small number of NFTs in the AD brainstem, such experiment may require significant numbers and sufficient volume of precious frozen samples from the brainstem (at least midbrain and pontine tegmentum), which are not readily available.

### What triggers the increase of the proportion of 3R tau-positive neurofibrillary changes with disease progression?

What are the possible mechanisms that regulate isoform-dependent deposition of tau in each neuron? If progressive increase of 3R tau in Alzheimer-type NFT mirrors relative abundance of 3R tau molecule or of its production, it is expected that the amount of mRNA is progressively upregulated in relevant areas. Although data on the brainstem have not been reported, mRNA of 3R tau is not upregulated in AD brains and in normal controls [8, 15, 26, 36, 58]. This raises a possibility that some post-translational modifications of 4R and 3R tau and their interactions may be responsible for such progressive representation of 3R tau over 4R tau. For example, in vitro assembly of 4R tau is significantly decreased by coincubation with 3R tau [1]. Another in vitro study has demonstrated that 4R and 3R tau monomers can grow onto the 4R/3R tau filaments and/or 3R tau filaments, while only 4R tau monomers can grow onto 4R tau filaments [12]. Do the aggregates composed of 4R and 3R tau exhibit corresponding immunoreactivity to both 4R and 3R tau? If an excess of one isoform over the other in the aggregates results in conformational alteration of tau molecules, epitope representation of each isoform could be skewed as if one of the two were absent, even if it is still integrated in the aggregates. Moreover, recent studies on mass spectrometric analyses of sarkosyl-insoluble and protease-resistant tau filaments from human brain with AD and cryo-electron microscopic analyses demonstrated that the protease-resistant cores of tau filaments contained abundant residues of the exon 11–12, which is proposed to adopt a combined cross- β/ β-helix structure with two identical protofilaments, as well as over a dozen of residues on the C-terminal side of exon 10 (4R tau) and exon 9 (3R tau), which exhibit unsharpened density on cryo-electron microscopy like a less ordered β-sheet [14, 48]. These results were compatible with the peptides identified in earlier studies, although there were some variations in the residue length [30, 57]. Still, it is unknown whether these C-terminal fragmentation of tau filaments occurs physiologically to the neurofibrillary changes in vivo. Even if the protease-resistant cores of tau filament are fundamental structures for neurofibrillary changes, how they are related to tau isoforms remains to be clarified because epitopes specific to tau isoforms were not included in the protofilament, a principal constituent of the protease-resistant cores. Because representation of each isoform is modified by aggregation, conformation and fragmentation, which epitopes of tau isoforms are involved in different tau filaments in the human brains may be crucial.

By taking advantage of the dual visibility of quantum dot (fluorescent nanocrystal identifiable also on electron microscopy) used as immunolabeling, we developed a method to correlate fluorescent microscopic images with their exact counterpart on immunoelectron microscopy [33, 49, 55]. This approach will clarify how either 4R or 3R tau epitope is involved in the formation of tau filaments at different stages of their formation in vivo. As double-immunofluorolabeling for 4R and 3R tau successfully demonstrated relationship between the isoforms at light microscopy level, we are now trying to improve this immunoelectron microscopy so that two different epitopes for 4R and 3R tau are visualized in relation to tau filaments of different morphologies and stages. It is expected that in vivo relation between 4R and 3R tau will be better understood if this double-labeling immunoelectron microscopy is successful for tau filaments.

### Discrepancies in the topographical distribution of tau and Aβ deposition

In the present study, the neurofibrillary changes were present without nearby Aβ in 43% of the cases (Table 1). Such discrepancy between tau and Aβ deposition is attracting an increasing attention [3, 7, 9, 13]. Regional distribution of tau [41] and that of Aβ [27, 51] in the brainstem have also been described. Because these had been described separately, it was not clear how tau and Aβ deposits are spatially related. The present study is the first that compared distribution of tau and Aβ deposits directly in neighboring sections of the same cases at different stages of disease progression (Figs. 2, 5, 6). The neurofibrillary changes were frequent in the ventral half of the PAG (Fig. 5a, b, open arrows), while the Aβ deposition was frequent in its dorsal half (Fig. 6l, solid arrow). Although there was an increase in the proportion of 3R tau-positive midbrain neurofibrillary changes with advancing neuritic plaque score (Fig. 3a *h, n*, b *h*), Aβ deposition was not directly linked to this increase of neurofibrillary changes, because their distributions were discrepant in the brainstem (Figs. 2, 5, 6). The most striking example of such discrepancy was detected in a case of centenarian (102 years old, case 21, Table 1), which showed a clinical history of dementia, 3R tau-dominant brainstem NFTs, NFT stages V/VI, but neuritic plaque score 0 and the absence of Aβ deposition at the midbrain and pons (Fig. 6k). Taken together, our observations suggested that the tau deposition and the progressive dominance of 3R tau-positive lesions may occur independently of Aβ deposition in the human brainstem at least to some extent.

## Conclusions

In conclusion, we have shown that a progressive increase in the proportion of 3R tau-positive lesions is extended to brainstem lesions as a fundamental to the pathogenesis of AD. The topographical differences between tau and Aβ deposits suggested that the formation of neurofibrillary changes and the increase in the proportion of 3R tau-positive lesions occur independently of regional Aβ deposition in the brainstem. In the future, ultrastructural localization of tau isoforms in relation to their filaments may clarify how changing proportion of isoforms are related to different stages of neurofibrillary changes, undertaking evolutionary changes from pretangles to ghost tangles.

## Additional files

Additional file 1: This file contains additional description of the image acquisition, and concomitant Lewy pathology of the investigated cases. The file also contains the results of additional statistical studies, including the regional difference in the proportion of 3R tau positive neurofibrillary changes on the same horizontal levels of the brainstem. (DOCX 25 kb)

Additional file 2: Figure S1. Quenching of autofluorescence after Sudan Black B treatment. Autofluorescence of intraneuronal lipofuscin is known to be prominent in the brainstem when quenching treatment is not performed. To clarify the effect of Sudan Black B treatment, we measured fluorescence emission spectra of intraneuronal lipofuscin on formalin-fixed, paraffin-embedded midbrain sections using Zeiss LSM780 lambda mode with or without the Sudan Black B treatment. The fluorescence spectrum of lipofuscin with excitation at 488 nm was broad and gently sloping (a-e, emission peak at 591 nm). This very intense fluorescence with broad spectrum agreed with the preceding studies on the fluorescence of lipofuscin. Sudan Black B treatment eliminated this autofluorescence of the lipofuscin (f-j, adjacent section). The fluorescence spectrum of lipofuscin did not overlap with the fluorescence spectra of Alexa 488- and Alexa 568-conjugated secondary antibodies labeling anti-4R tau antibody and RD3, respectively, on the pontine section, which underwent the Sudan Black B treatment (k-o). The small peak around 600 nm with excitation at 488 nm corresponded to colocalized Alexa 568 signal, which was blocked by the dichroic mirror on image capturing of the Alexa 488 signal. Therefore, we concluded that the autofluorescence of lipofuscin was effectively quenched by Sudan black treatment and did not affect the result of our study. Em: emission, Ex: excitation. (TIFF 7768 kb)

Additional file 3: Figure S2. Representative partial virtual slide images of the midbrain and pontine sections double immunofluorolabeled for 4R (green) and 3R tau (red), with colocalization (yellow). From top to bottom; case 7, 9, 18, in NFT stages I/II, III/IV, and V/VI, respectively. Insets in the low power field images (left row) correspond to the magnified images (right row). Bar = 200 μm and 50 μm, respectively. (TIFF 2019 kb)

Additional file 4: Figure S3. Comparable tau immunolabeling with two antibodies RD4 (monoclonal) and 4R tau (polyclonal). DAB immunohistochemistry using rabbit polyclonal anti-4R tau (a, 1:30,000 dilution) and mouse monoclonal RD4 (b, 1:1000 dilution) on adjacent hippocampal sections showed that the amount of immunolabeling with rabbit polyclonal anti-4R tau was equivalent to or slightly greater than the amount of immunolabeling with mouse monoclonal RD4. (TIFF 6926 kb)

Additional file 5: Figure S4. Predominance of RD3+ NFTs over RD4+ NFTs is shared between brainstem and hippocampus. To see whether the same trend of RD3 dominance for the neurofibrillary changes over RD4 is also demonstrated by the conventional DAB immunohistochemistry, we performed quantification of the RD3 and RD4 levels on representative adjacent pontine and hippocampal sections. After thresholding of the DAB labeling by RGB values, the counts and sizes of RD3 and RD4 were calculated on CellSens software (Olympus). RD3/RD4 ratio of total NFT counts and area on the pontine section were 6.00 and 4.49, respectively, indicating more intense deposition of RD3-positive neurofibrillary changes than RD4 (a, c, case 17). While the difference in the methods used make direct comparisons difficult, this observation of RD3 dominance agreed with our double-immunofluorolabeling of the same case using anti-4R tau antibody and RD3. RD3/RD4 ratio of total NFT counts and area on the hippocampal sections were 25.6 and 34.3, respectively. Thus, the dominance of 3R tau was also detected for the neurofibrillary changes in the hippocampal area of the same case (b, d, case 17). (TIFF 3761 kb)
