# Supplementary figures and images for: Brainstem tau pathology in Alzheimer’s disease is characterized by increase of three repeat tau and independent of amyloid β
Source: Acta Neuropathol Commun. 2018 Jan 3;6:1. doi: 10.1186/s40478-017-0501-1 (PMC5753447; doi:10.1186/s40478-017-0501-1)

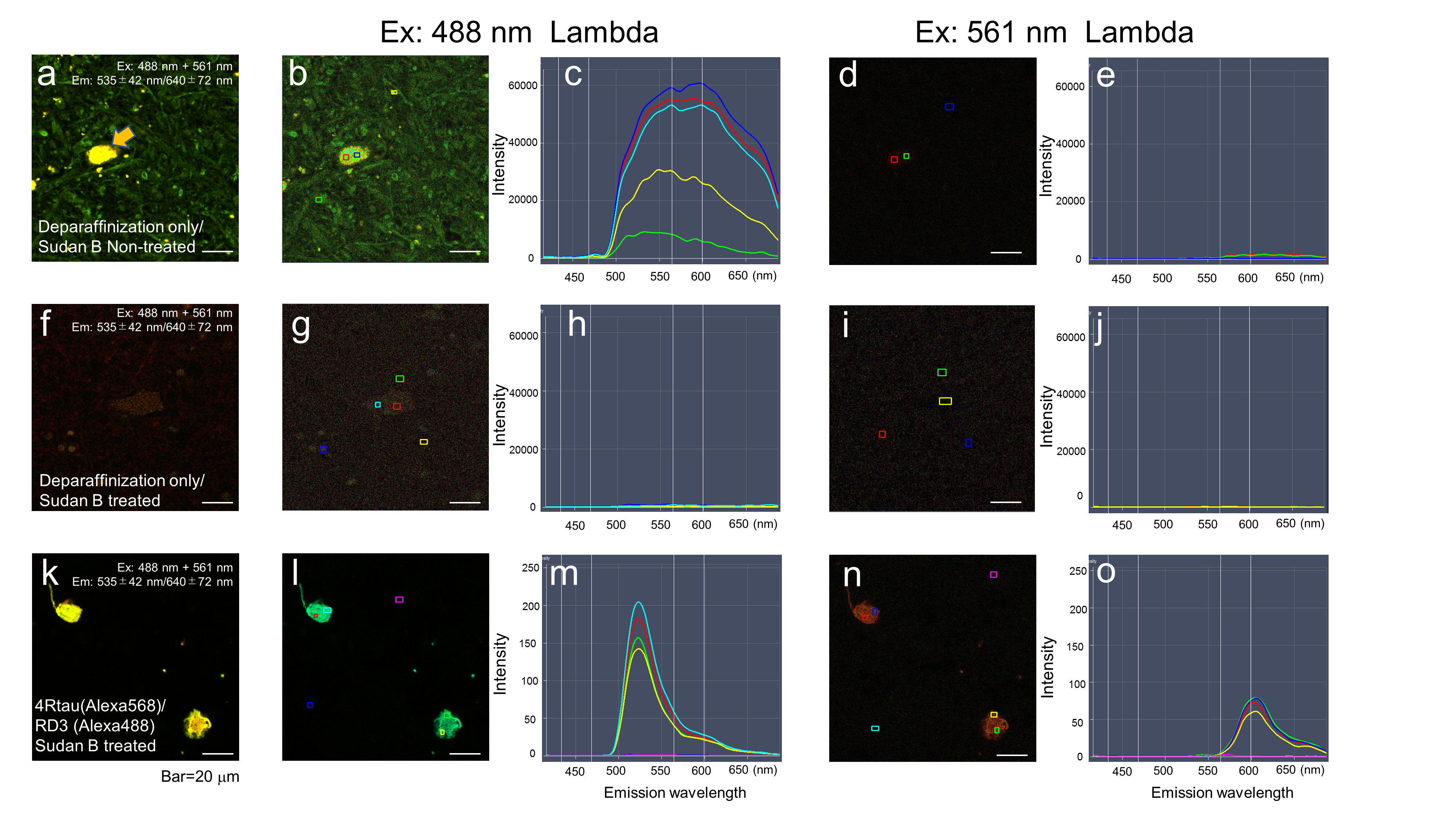

Supplement: Supplementary file 2 — Quenching of autofluorescence after Sudan Black B treatment. Autofluorescence of intraneuronal lipofuscin is known to be prominent in the brainstem when quenching treatment is not performed. To clarify the effect of Sudan Black B treatment, we measured fluorescence emission spectra of intraneuronal lipofuscin on formalin-fixed, paraffin-embedded midbrain sections using Zeiss LSM780 lambda mode with or without the Sudan Black B treatment. The fluorescence spectrum of lipofuscin with excitation at 488 nm was broad and gently sloping (a-e, emission peak at 591 nm). This very intense fluorescence with broad spectrum agreed with the preceding studies on the fluorescence of lipofuscin. Sudan Black B treatment eliminated this autofluorescence of the lipofuscin (f-j, adjacent section). The fluorescence spectrum of lipofuscin did not overlap with the fluorescence spectra of Alexa 488- and Alexa 568-conjugated secondary antibodies labeling anti-4R tau antibody and RD3, respectively, on the pontine section, which underwent the Sudan Black B treatment (k-o). The small peak around 600 nm with excitation at 488 nm corresponded to colocalized Alexa 568 signal, which was blocked by the dichroic mirror on image capturing of the Alexa 488 signal. Therefore, we concluded that the autofluorescence of lipofuscin was effectively quenched by Sudan black treatment and did not affect the result of our study. Em: emission, Ex: excitation. (TIFF 7768 kb) [file 40478_2017_501_MOESM2_ESM.tif]

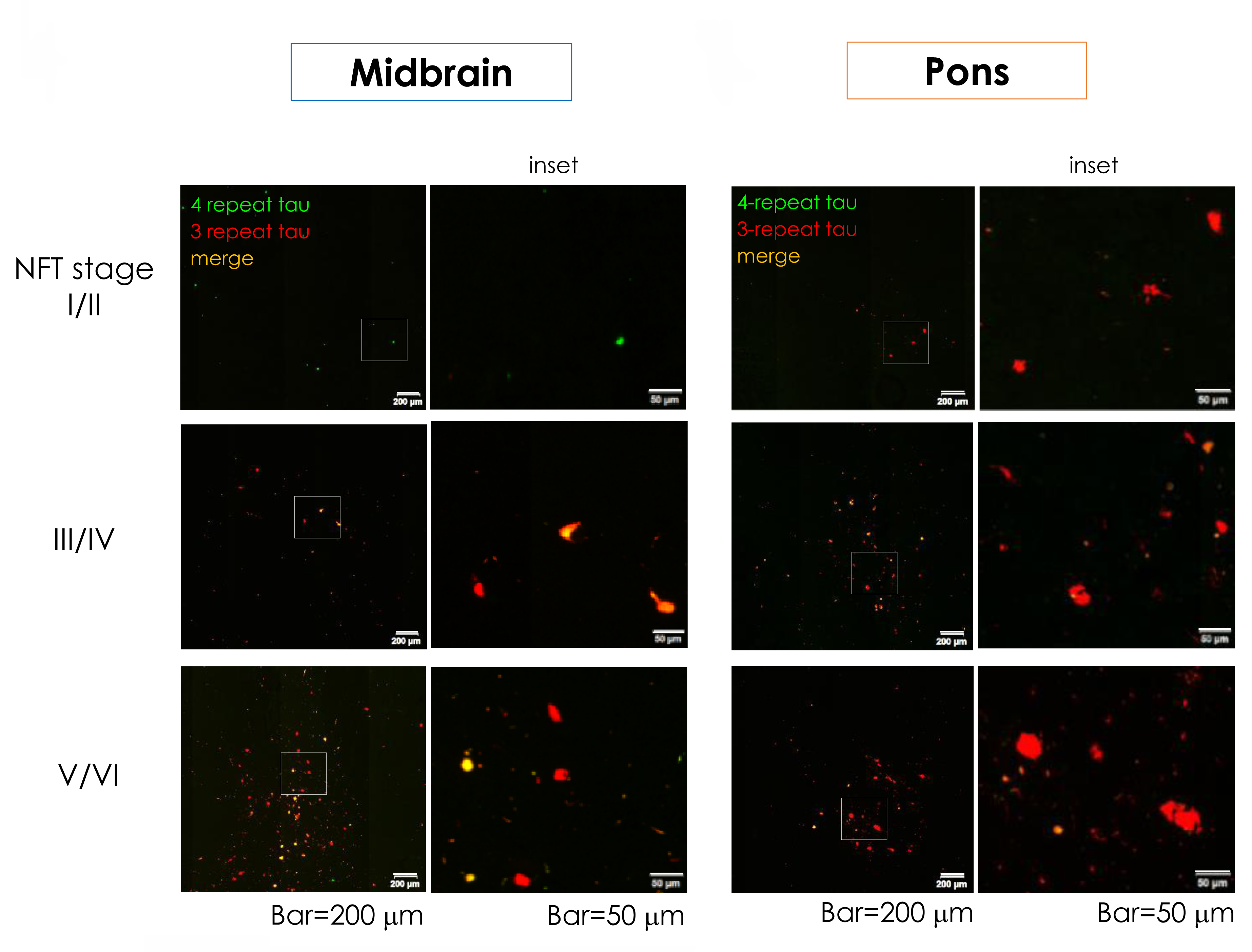

Supplement: Supplementary file 3 — Representative partial virtual slide images of the midbrain and pontine sections double immunofluorolabeled for 4R (green) and 3R tau (red), with colocalization (yellow). From top to bottom; case 7, 9, 18, in NFT stages I/II, III/IV, and V/VI, respectively. Insets in the low power field images (left row) correspond to the magnified images (right row). Bar = 200 μm and 50 μm, respectively. (TIFF 2019 kb) [file 40478_2017_501_MOESM3_ESM.tif]

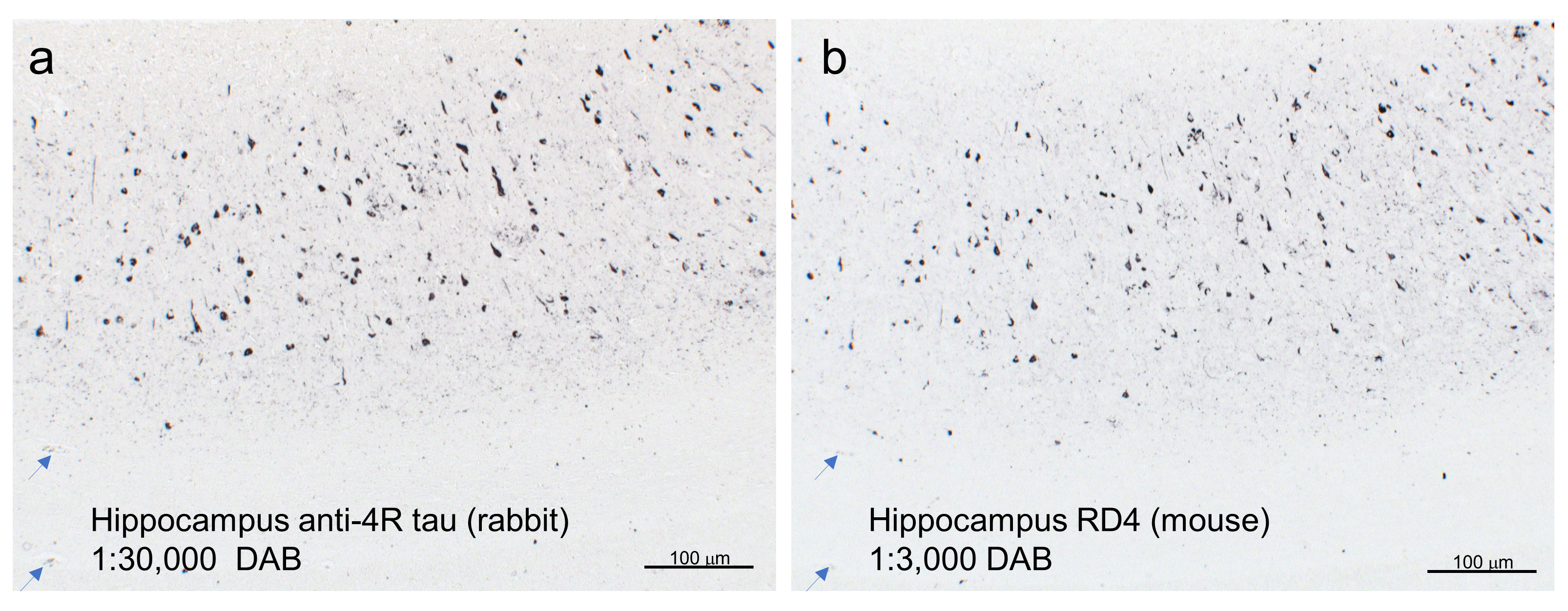

Supplement: Supplementary file 4 — Comparable tau immunolabeling with two antibodies RD4 (monoclonal) and 4R tau (polyclonal). DAB immunohistochemistry using rabbit polyclonal anti-4R tau (a, 1:30,000 dilution) and mouse monoclonal RD4 (b, 1:1000 dilution) on adjacent hippocampal sections showed that the amount of immunolabeling with rabbit polyclonal anti-4R tau was equivalent to or slightly greater than the amount of immunolabeling with mouse monoclonal RD4. (TIFF 6926 kb) [file 40478_2017_501_MOESM4_ESM.tif]

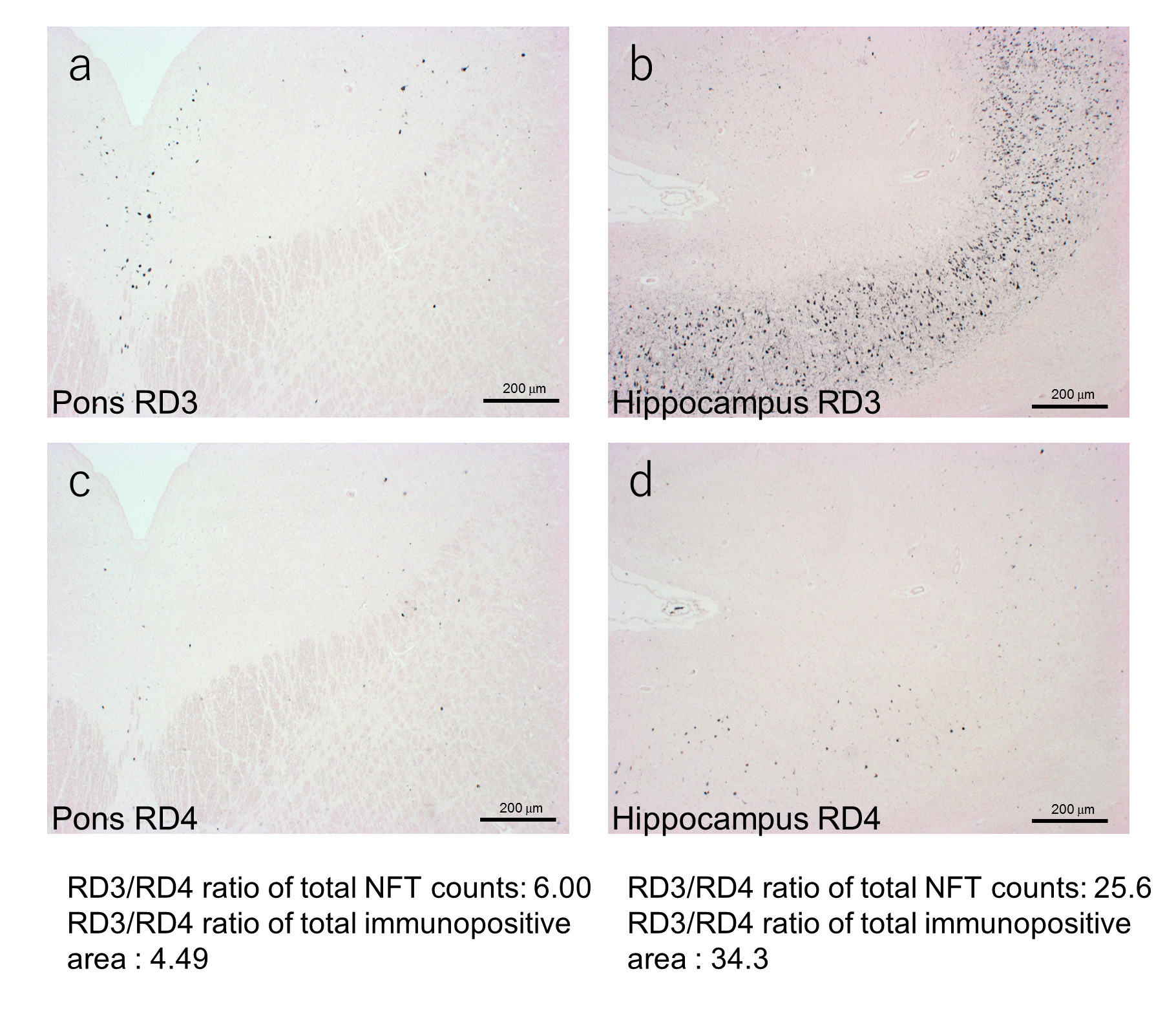

Supplement: Supplementary file 5 — Predominance of RD3+ NFTs over RD4+ NFTs is shared between brainstem and hippocampus. To see whether the same trend of RD3 dominance for the neurofibrillary changes over RD4 is also demonstrated by the conventional DAB immunohistochemistry, we performed quantification of the RD3 and RD4 levels on representative adjacent pontine and hippocampal sections. After thresholding of the DAB labeling by RGB values, the counts and sizes of RD3 and RD4 were calculated on CellSens software (Olympus). RD3/RD4 ratio of total NFT counts and area on the pontine section were 6.00 and 4.49, respectively, indicating more intense deposition of RD3-positive neurofibrillary changes than RD4 (a, c, case 17). While the difference in the methods used make direct comparisons difficult, this observation of RD3 dominance agreed with our double-immunofluorolabeling of the same case using anti-4R tau antibody and RD3. RD3/RD4 ratio of total NFT counts and area on the hippocampal sections were 25.6 and 34.3, respectively. Thus, the dominance of 3R tau was also detected for the neurofibrillary changes in the hippocampal area of the same case (b, d, case 17). (TIFF 3761 kb) [file 40478_2017_501_MOESM5_ESM.tif]
