# Supplementary material for: Brainstem tau pathology in Alzheimer’s disease is characterized by increase of three repeat tau and independent of amyloid β
Source: Acta Neuropathol Commun. 2018 Jan 3;6:1. doi: 10.1186/s40478-017-0501-1 (PMC5753447; doi:10.1186/s40478-017-0501-1)
Supplement: Supplementary file 3 [file 40478_2017_501_MOESM1_ESM.docx]

Additional file 1

Number of snapshots and total areas captured in CENSUS study of 4R and 3R tau double immunofluorescence

The virtual slide system VS120 captures serial fluorescent snapshots, and the adjacent snapshot is arranged to overlap with the border of the proximate snapshot to stitch the images together.  The resolution of each snapshot is 1376 pixels (horizontal)×1038 pixels (vertical) at 0.645 μm/pixel with 10×objective lens.  After subtracting the overlapping margins, the area per snapshot reduces to approximately 1138 pixels×834 pixels (approx. 0.398 mm2/snapshot).  We captured the entire tectum and tegmentum of each sections for our study (Fig. 1a, right).  10 sets of images (5 vertical planes in 2 separate fluorescence channels) were captured for each section.

The number of snapshot images captured was 8,804±891 and 4,240±1,340 per section (mean±SD) of the midbrain and pons, respectively.  This number of snapshots did not differ significantly between cases with different NFT stages (p=0.455 and p=0.820 for midbrain and pons, respectively, ANOVA).

The number of snapshots of the midbrain was significantly larger than that of the pons (p=2.05×10-12, paired t-test).  This is explained by the difference in the original sizes of the entire tectum and tegmentum between the midbrain and pons.  The surface area captured of the midbrain and pons was 3.51±0.355 cm2 and 1.69±0.53 cm2 per section (mean±SD), respectively.  The difference in the areas between midbrain and pons did not affect the result of our study because we did not perform direct comparison of the degrees of neurofibrillary pathology between midbrain and pons.  

The total sum of the snapshots captured for this study was 300,860 (approx. 1,197 cm2); 203,280 (approx. 809 cm2) for the midbrain and 97,580 (approx. 388 cm2) for the pons.  The target surface area (1/10 of total captured area) was approximately 120 cm2 in total; 80.9 cm2 for the midbrain and 38.8 cm2 for the pons.  Data mining of immunolabels from such a broad area was made possible by virtual slide capturing of sectional images with standardized condition at high resolution, and it is the strength of our CENSUS method.

 

Concomitant Lewy pathology

  Frequent association of Alzheimer’s pathology and Lewy bodies has been reported in AD cases.  Concomitant Lewy pathology was observed in case 1 (brainstem predominant), 9 (brainstem predominant), 13 (transitional) and 23 (neocortical), according to McKeith guidelines for the evaluation of Lewy body distribution and frequency [37].  There were no significant differences between cases with and without Lewy pathology in the means of the count of neurofibrillary changes or the percentages of tau isoforms (Welch two sample t-test, p>0.05).

Estimation of odds ratio for the dominance of 3R tau over 4R tau with advancing NFT stage

  The greater proportion of 3R tau than that of 4R tau was detected for the neuropil threads (NTs) and neurofibrillary tangles (NFTs) in 54 % (25/46) and 67 % (31/46) of the midbrain and pontine sections, respectively (Table 1, > indicates that the proportion of 3R tau is greater than that of 4R tau, < indicates that the proportion of 4R tau is greater than that of 3R tau, on each section).  That was: 25 % (4/16), 69 % (11/16), 71 % (10/14) for the NTs, and 44 % (7/16), 75 % (12/16), 86 % (12/14) for NFTs, of the midbrain and pontine sections from the cases with the cortical NFT stages I/II, III/IV, V/VI respectively (Table 1).  We estimated the odds ratio (OR) and 95 % confidence intervals (CIs) for the percentages of sections with greater proportion of 3R tau than 4R tau with the advancement of cortical NFT stages (from I/II, III/IV to V/VI) by a univariate binomial logistic regression model.  The estimated OR and 95 % CIs for the increase of 3R dominant sections with advancing cortical NFT stages were 2.84 (1.30-6.92) and 2.94 (1.27-7.88) for the NTs and NFTs, respectively.  This indicted that the advancement of cortical NFT stage was significantly associated with greater proportion of 3R tau than that of 4R tau in the neurofibrillary changes of the midbrain and pons.

3R tau dominance of midbrain NFT in the group of advanced age, NFT stage and lowest brain weight

  Since we have observed an increasing trend of the proportion of 3R and/or 4R/3R both positive midbrain NFTs with advancing age and Alzheimer-related cortical pathologies (Fig. 3B f-j), we next assessed whether there was a difference in the means of the proportion of tau isoforms in the advanced groups.  We performed paired t-test with Bonferroni correction in cases with very old age (age above 91, n=7), NFT stages V/VI (n=7), CERAD score 3 (n=6), and brain weight below 1200 g (n=8), for the difference of the proportion of 3R and 4R tau positive midbrain NFTs.  The proportion of 3R tau positive midbrain NFT was significantly higher than that of 4R tau in the cases with very old age, NFT stages V/VI, and brain weight below 1200 g (p=0.0017, 0.033, 0.0017, respectively, paired t-test with Bonferroni correction), but not CERAD score 3 (p=0.073).

  On the other hand, midbrain and pontine NTs did not show the similar trend of 3R tau dominance in the advanced groups (paired t-test with Bonferroni correction, p=1, 1, 1, 0.94, and p=0.17, 1, 0.50, 0.23, respectively, midbrain and pontine NTs, for very old age, NFT stages V/VI, CERAD score 3 and brain weight below 1200g).  Taken together, midbrain NFT in the cases of advanced age, NFT stages V/VI and low brain weight, showed 3R tau dominance, while midbrain and pontine NTs did not.

Regional difference in the proportion of 3R tau positive neurofibrillary changes

  Since it was revealed that the 3R tau positive neurofibrillary changes increase with advancing age and Alzheimer-related cortical pathology at the midbrain, we next aimed to clarify whether there was a regional difference in the proportion of 3R tau positive lesions within the midbrain sections.  For this purpose, the midline region and lateral tegmental region (left side) of the midbrain were extracted from the virtual slide images (Fig. 1a, right).  The lateral tegmental region encompassed all part of substantia nigra on the section.  Likewise, the midline region and lateral region (left side) of the pons were also extracted from the virtual slide images for comparison (Fig. 1a, right).  These parts also underwent same procedure of quantitative analysis as described above.  At the midbrain, the paired t-test revealed significantly higher means of the proportion of 3R tau positive NFTs at the midline region than the lateral tegmental region (paired t-test, p= 0.0042).  At the pons, however, the paired t-test did not reveal significant difference between the midline region and the lateral region (paired t-test, p=0.15).  Taken together, these findings indicated that the midline region of the midbrain showed higher proportion of 3R tau positive NFTs than the lateral tegmental region represented by substantia nigra.  The distribution of tau isoform was shown to be inhomogenous between different nuclei, even when they are on the same horizontal level at the midbrain.
